# Supplementary material for: Hierarchical porous materials made by stereolithographic printing of photo-curable emulsions
Source: Sci Rep. 2021 Nov 16;11:22316. doi: 10.1038/s41598-021-01720-6 (PMC8595381; doi:10.1038/s41598-021-01720-6)
Supplement: Supplementary file 1 — Supplementary Information. [file 41598_2021_1720_MOESM1_ESM.docx]

**Supplementary Information: Hierarchical porous materials made by stereolithographic printing of photo-curable emulsions**

Nicole Kleger, ^1^  Clara Minas, ^1^  Patrick Bosshard, ^1^  Iacopo Mattich^1,2^, Kunal Masania, ^1,2^* André R. Studart ^1^*

^1^ Complex Materials, Department of Materials, ETH Zurich, 8093 Zurich, Switzerland

^2^ Soft Materials, Department of Materials, ETH Zurich, 8093 Zurich, Switzerland

^3^ Current address: Shaping Matter Lab, Faculty of Aerospace Engineering, Delft University of Technology, Kluyverweg 1, 2629 HS Delft, Netherlands

*corresponding authors: [k.masania@tudelft.nl](mailto:k.masania@tudelft.nl), [andre.studart@mat.ethz.ch](mailto:andre.studart@mat.ethz.ch)

**Content:**

Influence of monomer ratio and nanoparticle content on the droplet size

Rheological behavior of Pickering emulsion resins

Polymerization behaviour of Pickering emulsion resins

Stereolithographic printing of high-fidelity structures

Elastic modulus of hierarchically porous lattices with tuneable strut microporosity

Maximum compressive strength of hierarchically porous lattices

Pyrolysis and sintering of printed hierarchical structures

SLA printed object with graded porosity

Adsorption properties of hierarchical porous structures

Pseudo-second order kinetic adsorption fitting of methylene blue adsorption

Surface area of as-printed and ceramic structures

Supplementary Figures 1-12

**Influence of monomer ratio and nanoparticle content on the droplet size**

In addition to the H_2_O content, the PUA monomer and the SiO_2_ particles may also affect the type of emulsion formed and the droplet size achieved. Varying the concentration of PUA relative to the other monomer (HDDA) in the emulsion influences significantly the pore size distribution of the printed structures (Supplementary Figure 1). For emulsions with a fixed water content of 45 vol% (40 wt%), we found that the average pore size decreases from 1.97 ± 1.54 µm to 0.50 ± 0.26 µm by increasing the PUA concentration from 0 to 10 wt% relative to the mass of HDDA. Such decrease in average droplet size is accompanied by a significant narrowing of the pore size distribution. When the PUA concentration is further increased above 28 wt%, a transitional phase inversion from a water-in-oil to an oil-in-water emulsion is observed. Such transition suggests that the PUA monomer reduces the wettability of the modified silica particles in the oil phase. This favours the displacement of the interfacially-adsorbed particles towards the water phase, which can eventually cause the emulsion to phase invert at sufficiently high PUA concentrations. This interpretation is in line with previous reports in the literature^21, 39^ and provides a rationale for the monomer composition selected to tailor the wetting and self-assembly behaviour of the particles within the emulsion.

To investigate the possible effect of the silica concentration on the emulsion structure, we prepared formulations with 10.9 – 27.1 wt% SiO_2_ and fixed PUA and H_2_O concentrations of 10 wt% (with respect to HDDA) and 40 wt% (44.9 vol%), respectively. Increasing the SiO_2_ concentration was found to improve the monodispersity of the pore size, while only slightly decreasing the mean droplet and pore sizes (Supplementary Figure 2). This is in line with an expected coalescence of droplets due to reduced particle surface coverage of the water-oil interface at lower silica concentrations. The initially < 500 nm droplets generated under tip sonication are expected to show a particle surface coverage below a monolayer (< 90%) if the silica nanoparticle concentration is lower than 20 wt%. The incomplete surface coverage will hence inevitably result in droplet coalescence and hence broader size distribution.


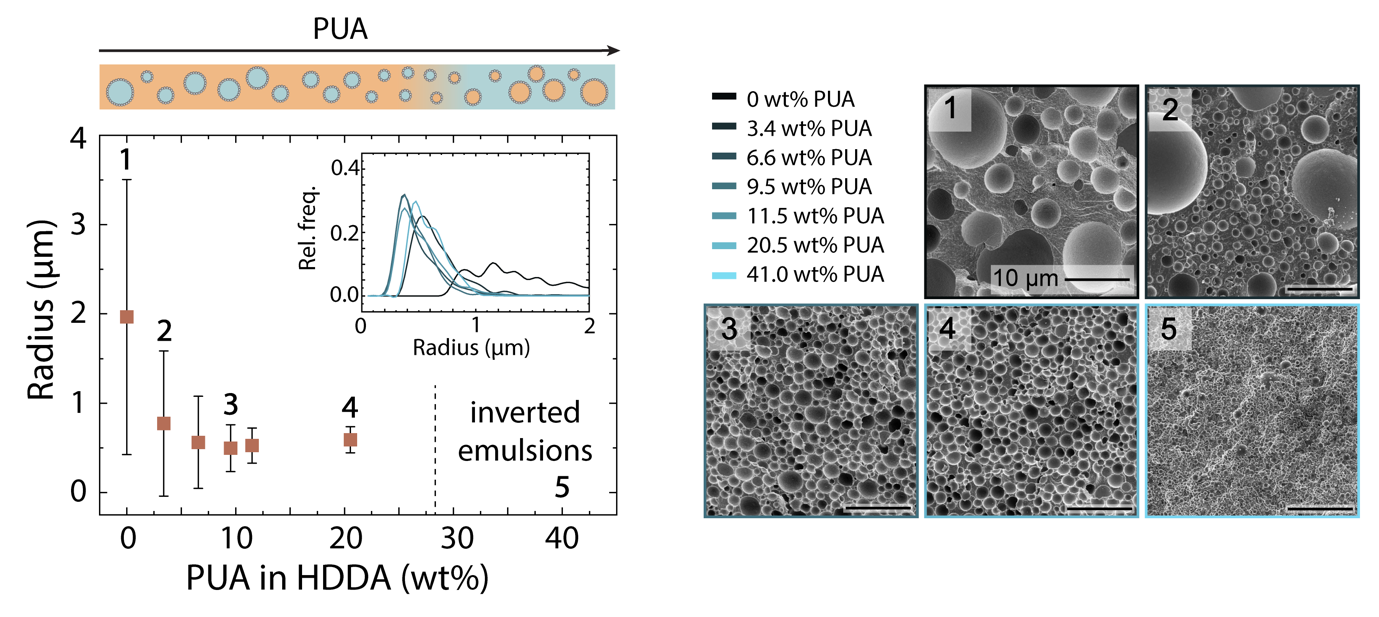


**Supplementary Figure 1.** Effect of the concentration of the PUA monomer on the type of emulsion and the size of droplets achieved upon mixing (left) and on the structure of the cured resin after drying and curing (right). The corresponding droplet size distributions are shown as an inset. Scale bar: 10 µm.

**
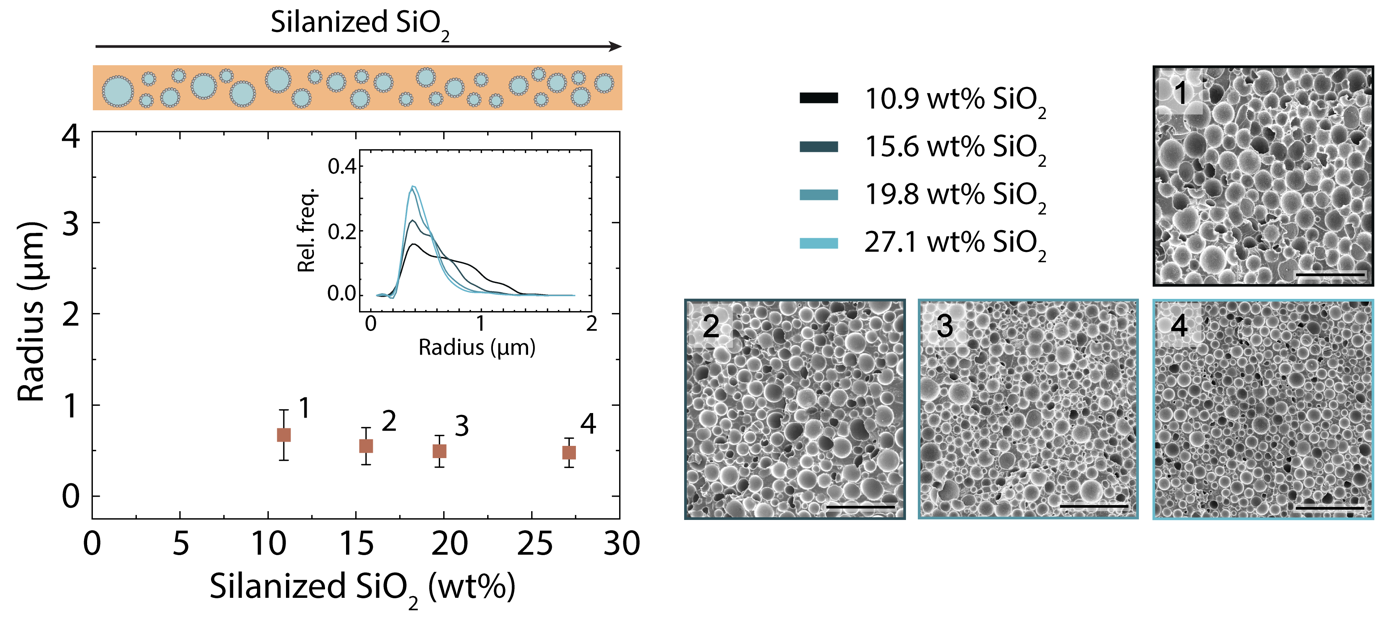
**

**Supplementary Figure 2.** Influence of the concentration of the SiO_2_ nanoparticles on the size of droplets achieved upon emulsification (left) and on the structure of the cured resin after drying (right). The corresponding distributions of the droplet sizes are shown as an inset. Scale bar: 10 µm.

**Rheological behavior of Pickering emulsion resins**

Full rheological characterization of emulsion resins without (Supplementary Figure 3a) or with 10 wt% PUA (Supplementary Figure 3b) was performed for water contents of 0 vol%, 29 vol% or 45 vol%. All measurements were taken on an Anton Paar MCR 302 rheometer (Anton Paar GmbH) using a setup comprising of a sand-blasted 25 mm parallel plate positioned at a 1 mm gap size. Amplitude sweeps were performed at logarithmically increasing shear stresses from 0.0001 to 100 Pa at a constant frequency of 1 rad/s to obtain the storage modulus *G’* and loss modulus G’’ (Supplementary Figure 3, I). Additionally, strain-controlled frequency sweeps (Supplementary Figure 3, II) were performed by logarithmically increasing the angular frequency from 0.1 to 500 rad/s at a constant shear strain of 0.01%. The resulting *G’’* values were used to calculate the dynamic viscosity of the emulsions (Supplementary Figure 3d), which is given by $G^{''}/\omega$, where $\omega$ denotes the angular frequency. The reported high-frequency viscosities were calculated for angular frequencies higher than 100 rad/s, above which the dynamic viscosity was found to be stable. Finally, steady-state measurements were performed by logarithmically increasing shear stresses from 0.01 to 100 Pa. The yield stress (Supplementary Figure 3d) was taken as the point at which the shear strain suddenly increases in the strain-stress curves obtained from the steady-state measurements. All measurements were performed at 25 °C.


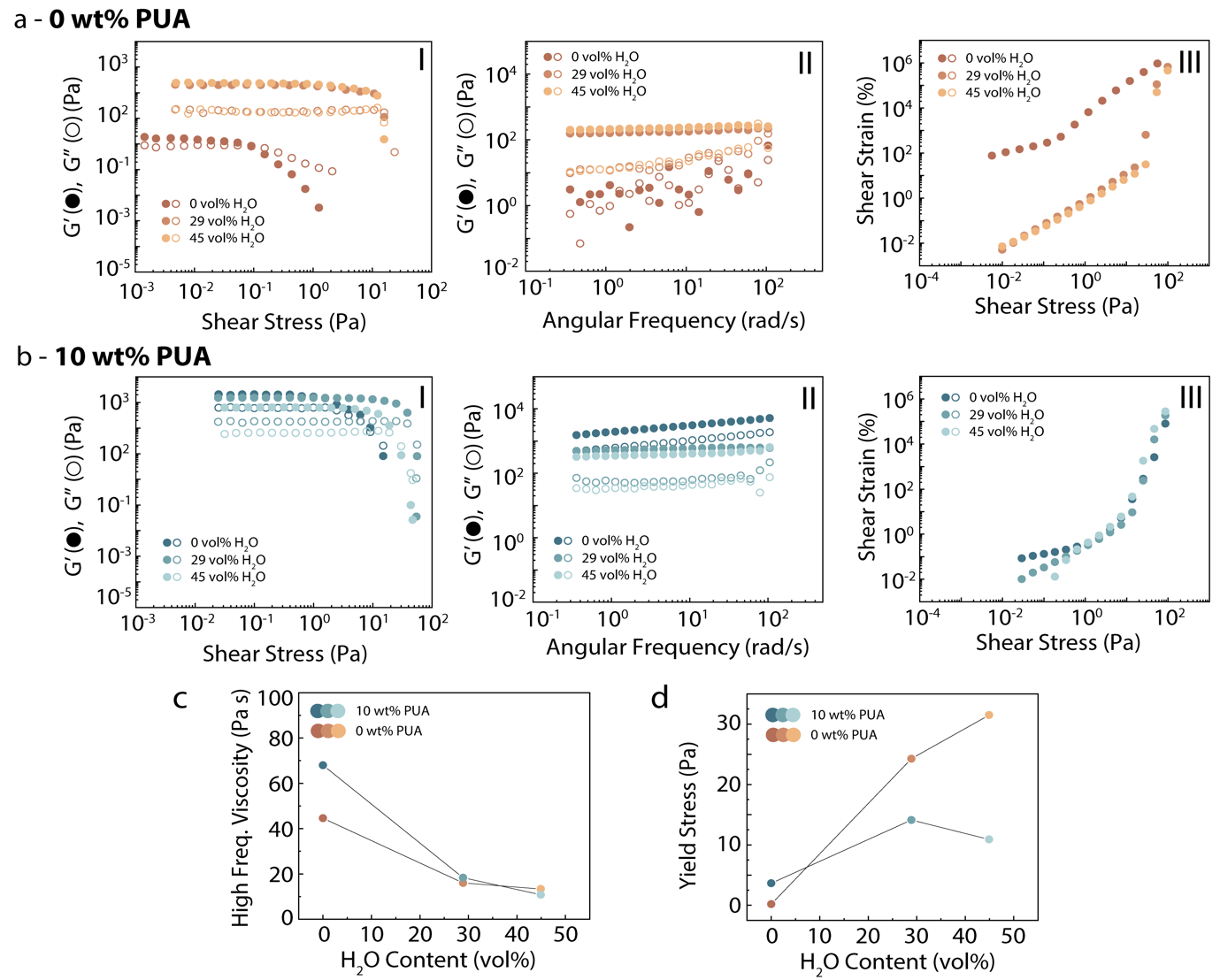


**Supplementary Figure 3.** Rheological behavior of emulsions prepared with water contents of 0%, 25% and 50% vol, (a) with and (b) without the addition of PUA monomer, consisting of an amplitude sweep (I), frequency sweep (II) and steady state measurement (III). (c) High-frequency dynamic viscosity and (d) yield stress of emulsions with and without PUA as a function of the water content.

**Polymerization behaviour of Pickering emulsion resins**

To identify emulsion formulations and printing conditions that lead to high-fidelity porous structures, we first analyse the effect of compositional and processing parameters on the polymerization behaviour of the reactive layer. Using Lambert-Beer’s law to quantify the decay in light intensity along the build direction, the effect of the initiator and UV-blocker concentrations on the measured thickness of the polymerized layer can be predicted reasonably well, allowing us to gain insights into the physical mechanisms underlying the polymerization process (Figure 2a,b, main text).

According to the Lambert-Beer mode, the thickness of the polymerized layer ($z_{p}$) depends on the light dose (*D*) as follows^24^: $z_{p}=h_{a}*\ln\left( \frac{D}{D_{c}} \right)$, where $h_{a}$ is the light penetration depth and $D_{c}$ is the critical dose of light required to initiate polymerization. Single-layer printing experiments were performed to verify the validity of this analytical expression in predicting the polymerization behaviour of photo-curable emulsions with selected concentrations of initiator and UV-blocker (Figure 2a,b). In these experiments, the light dose *D* (units of energy) is deliberately varied by changing the exposure time at a constant light intensity (units of power).

The results show that the theoretical model predicts the effect of the initiator and UV-blocker concentrations on the measured thickness of the polymerized layer reasonably well, allowing us to gain insights into the physical mechanisms underlying the polymerization process. By plotting the measured thickness as a function of the log(*D*), we can directly obtain the penetration depth $h_{a}$ and the critical dose $D_{c}$ from the slope and the x-axis intercept of the theoretical fits, respectively. For compositions with fixed UV-blocker content, the analysis reveals that an increase in initiator concentration initially reduces the critical dose necessary for polymerization ($D_{c}$) while keeping the penetration depth ($h_{a}$) mostly unchanged (Figure 2a). If the nominal initiator concentration surpasses a threshold value of 0.5wt% (with respect to the monomer), no effect on the critical light dose is observed. These findings are in line with earlier reports^40, 41, 42^ and reflect the fact that a minimum concentration of initiator molecules need to be photo-activated to initiate the chain growth reaction. Our data suggest that this minimum amount of reactive molecules is reached for formulations with a nominal initiator content of at least 0.5 wt% combined with a light dose equal or higher than 3 mJ/cm^2^. Below this nominal initiator content, the number of reactive species can be increased by enhancing the light dose applied, which explains the observed correlation between $D_{c}$ and the amount of initiator at low nominal concentrations. Conversely, higher UV-blocker concentrations were found to decrease the penetration depth ($h_{a}$) without affecting so much the critical light dose $D_{c}$ (Figure 2b). This dependence qualitatively follows the inverse correlation between penetration depth and the concentration of light absorbing species expected from Lambert-Beer’s law.^43^

**Stereolithographic printing of high-fidelity structures**

Understanding the polymerization behaviour of the photo-curable emulsions enables the design of formulations suitable for printing cellular materials with programmable pore size and strut thickness, while minimizing the required light dose and printing time. We quantify the fidelity and the programmability of the stereolithographic process by printing specimens featuring large arrays of holes or pillars with pre-defined sizes along the build direction or within the plane of the substrate (x-y plane), respectively. Holes and pillars are used in these experiments as simplified negative and positive features analogous to pores and struts in a printed structure. These structures were printed with a constant light intensity of 20 mW/cm^2^ at varying illumination times of 2, 3 or 4 s per layer, resulting in doses of 40, 60 and 80 mJ/cm^2^, respectively. Experiments were carried out using an emulsion formulation containing the threshold initiator concentration of 0.5wt% and an UV-blocker content (0.075 wt%) that should lead to a nominal printed layer thickness of 50 µm.

Although light doses as low as 3 mJ/cm^2^ are enough to create a gelled polymerized layer, we experimentally observed that fully cured layers require doses of at least 40 mJ/cm^2^. For an applied light dose of 40 mJ/cm^2^, the size of holes printed along the build direction (*d_exp_*) were found to scale linearly with the input target values (*d_tar_*) with a slight offset *δ* (Supplementary Figure 3a). The offset might be attributed to local over-polymerization of the resin caused by the diffusion of initiator molecules into non-exposed areas. Such undesired effect is probably the reason for the inaccuracy observed for holes smaller than 500 µm and printed at the higher doses of 60 and 80 mJ/cm^2^. In this case, over-polymerization leads to holes with oval shapes that are much smaller than the target dimensions (*d_tar_*).

For the experiments with pillars, we found that the pillar size (*d_exp_*) scales linearly with the target value if *d_tar_* is at least 4 times larger than the thickness of a single polymerized layer (>200 µm, Supplementary Figure 3b). High light doses of 60 and 80 mJ/cm^2^ lead to over-polymerization and thicker dimensions compared to the target values, whereas thinner pillar sizes are obtained for the lowest light dose (40 mJ/cm^2^). Below the critical size of 200 µm, a poor correlation between experimental and target values is observed, likely due to the limited lateral resolution of the desktop printer used in the study.

Overall, these model experiments indicate that pore sizes and strut thicknesses down to 200 µm can be printed with high accuracy if the measured offsets between the experimental and target values (Figure 2c and Supplementary Figure 3) are taken into account in the geometrical design.


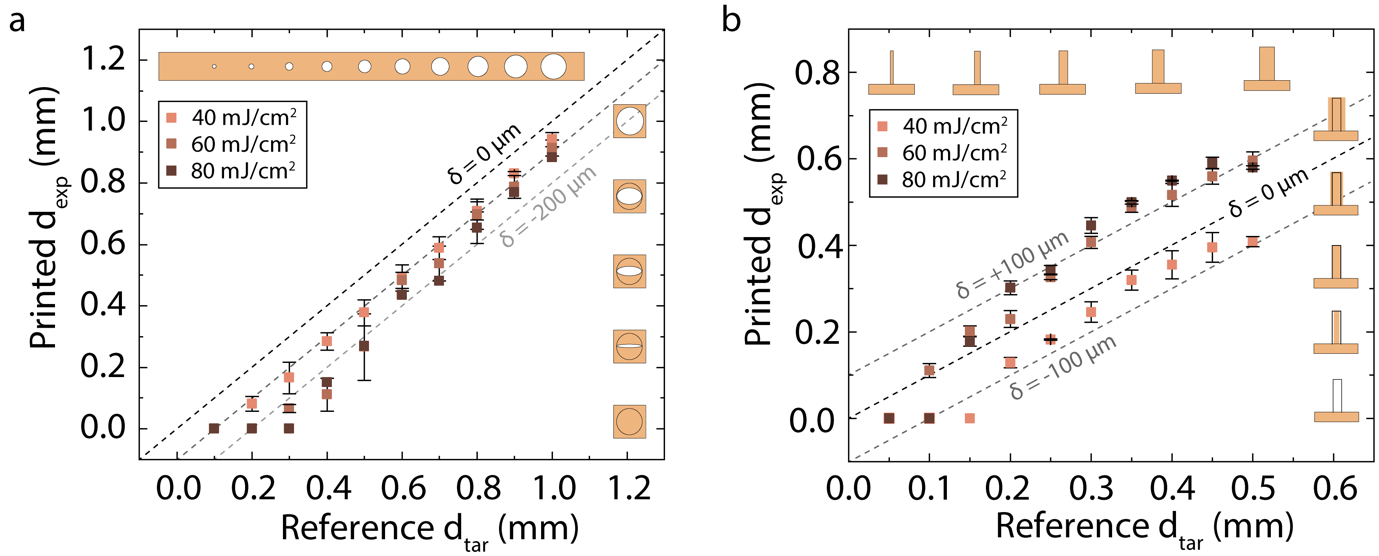


**Supplementary Figure 4.** Printing fidelity achieved for negative (holes) and positive (pillar) features using different illumination doses. Dose levels of 40, 60 and 80 mJ/cm^2^ were applied by using exposure times of 2, 3 and 4 s, respectively, at a fixed light intensity of 20 mW/cm^2^. The dashed line represents the perfect print, corresponding to a 1:1 translation of the print file to the printed object. Different values for the offset $\delta$ are included in the plots.

**Elastic modulus of hierarchically porous lattices with tunable strut microporosity**

In addition to changes in the strut thickness (main text), a second strategy to vary the relative density of hierarchical lattices consists in the incorporation of an increasing volume fraction of micropores in the struts up to 50 vol% while keeping their thickness constant at 0.5 mm (Supplementary Figure 4). Analogous to hierarchical structures with varying strut thickness, this approach enhances the mechanical efficiency of bending-dominated Kelvin lattices and promotes a stronger drop in elastic modulus of stretching-dominated octet lattices. The scaling exponent decreases from 2.77 to 1.48 for Kelvin structures and increases from 1.88 to 2.54 for octet lattices. These shifts in power exponents can be rationalized following the same arguments used to explain the behaviour of hierarchical structures displaying different strut thicknesses.


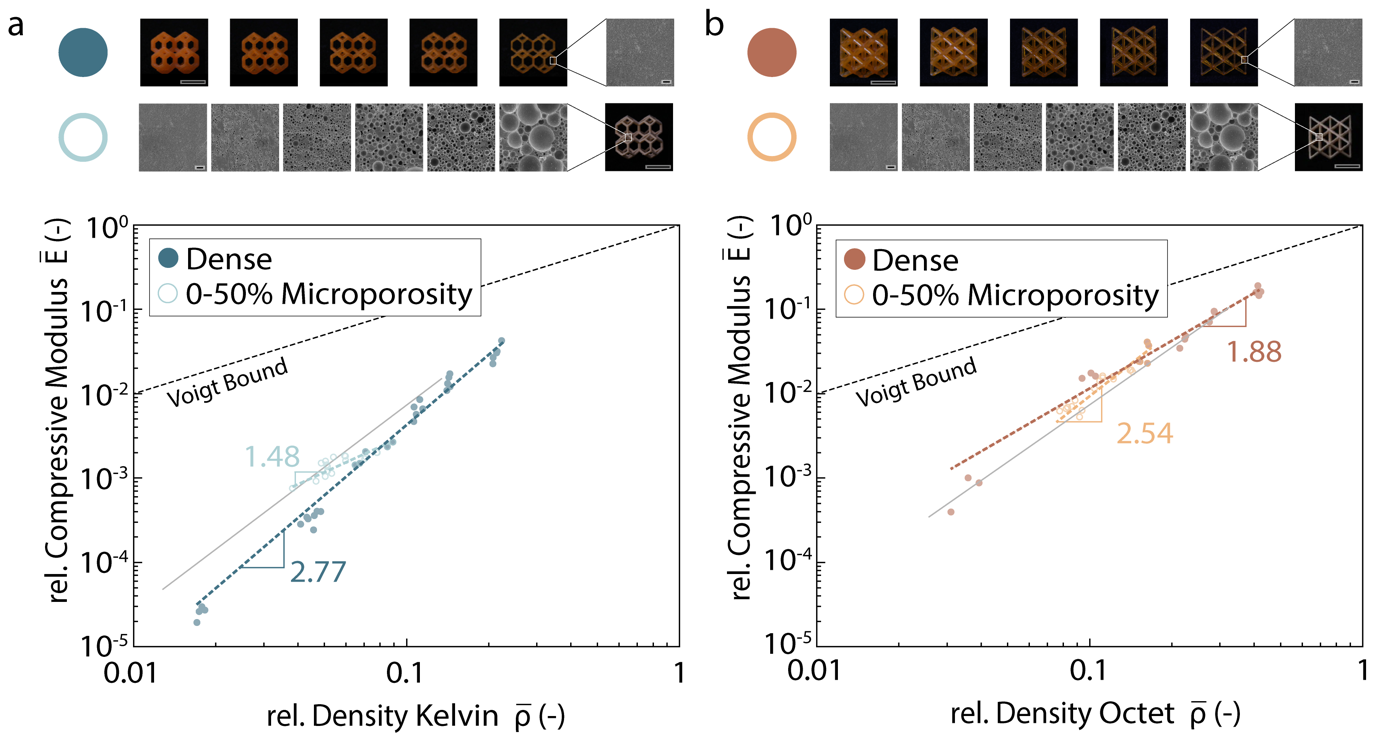


**Supplementary Figure 5.** Relative compressive modulus of (a) Kelvin and (b) octet lattices with dense struts of variable thickness (dense) compared to counterparts with fixed strut thickness (0.5 mm) and microporosity varies within the range of 0 – 50 vol% inside the struts. The coloured dashed lines show fittings obtained using the analytical model. The numbers next to such lines correspond to the power exponents. The Voigt bound is displayed as a black dashed line, whereas the full grey lines shown in (a) and (b) indicate the fittings obtained for the lattices with 45 vol% porous struts of varying strut thickness.

**Maximum compressive strength of hierarchically porous lattices**


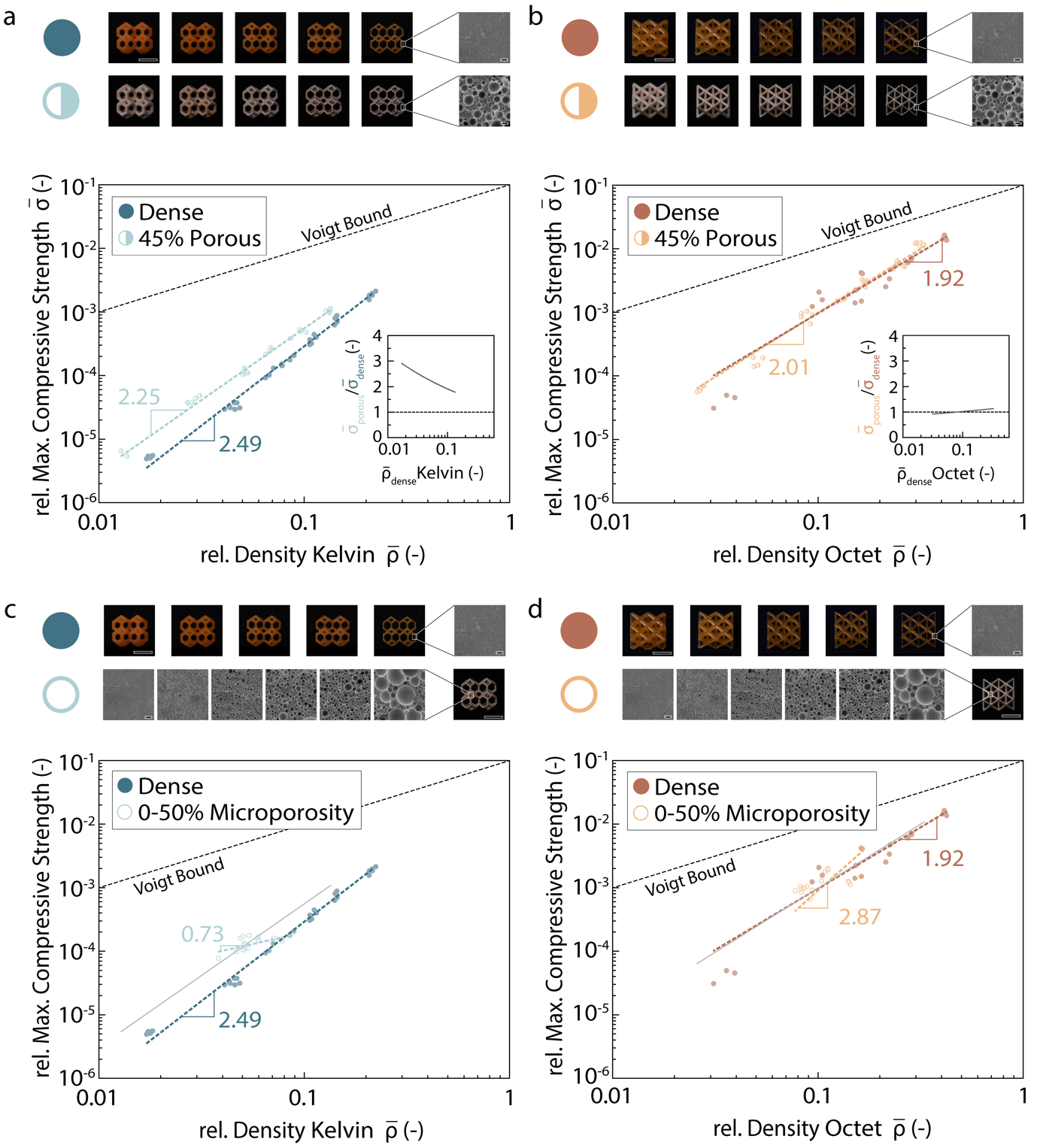


**Supplementary Figure 6.** Mechanical strength of Kelvin and octet hierarchical lattices with varying relative densities. (a,b) Relative compressive strength ($\bar{\sigma}$) of (a) Kelvin and (b) octet lattices with struts that are either dense or 45% porous. In these samples the relative density is changed by varying the strut thickness. The insets show the ratio between the relative maximum compressive strength for microporous and dense Kelvin lattices (${\bar{\sigma}_{porous}}/{\bar{\sigma}_{dense}}$) at varying relative densities of the overall structure ($\bar{\rho}$). (c,d) Relative compressive strength of (c) Kelvin and (d) octet lattices with dense struts of variable thickness (dense) compared to counterparts with fixed strut thickness (0.5 mm) but microporosity varying between 0 and 50 vol% inside the struts. The coloured dashed lines show fittings obtained using the analytical model. The numbers next to such lines correspond to the power exponents. The Voigt bound is displayed as a black dashed line, whereas the full grey lines shown in (c) and (d) indicate the fittings obtained for the lattices with 45 vol% porous struts of varying thickness. Scale bars: 5 mm in the macroscopic images of lattices (Kelvin and octet); 10 µm in the SEM close-ups of dried emulsions.

**Pyrolysis and sintering of printed hierarchical structures**

As-printed composites can be converted into inorganic hierarchical structures by pyrolysis of the organic phase followed by sintering of the remaining oxide phase. The processing conditions required for slow removal of the organic phase during calcination were evaluated by performing thermogravimetric analysis (TGA) of the as-printed composites (Supplementary Figure 6). The TGA data reveals that heating of the printed material in air results in two main mass loss processes between 50 and 500 °C. At lower temperatures in the range of 25-200 °C, the material loses about 18% of its initial weight, which is associated with the evaporation of residual physically and chemically bound water from the structure. Heating the sample further up to the temperature window 300-500 °C leads to two sequential drops in weight and an additional mass loss of 58%. These processes correspond to the thermal oxidation and removal of the polymer phase, after which the structure is finally ready for sintering.

The sintering behaviour of the calcined structures was investigated by measuring the dimensional changes of 45 vol% porous specimens upon heating up to 1100 °C and 1200 °C (Supplementary Figure 7a). Shrinkage of the structure starts already at 500 °C and continues at the dwell temperatures to reach values between 10 and 42% at the end of the sintering process. Importantly, the sintering time at high temperatures strongly affects the structure of the material at the microscale. SEM images of sintered samples indicate that the assembly of silica nanoparticles initially present on the walls of the microscale pores undergo extensive morphological changes during the heat treatment (Supplementary Figure 7b). At 1100 °C, the pore wall morphology changes within the first hour of sintering from a network of particles hold by interconnecting necks to a dense assembly of grains around closed pores. For longer sintering times, the spherical pores eventually transform into an open porous network with coral-like morphology. When sintered at the higher temperature of 1200 °C, this open network finally coalesces into a denser microstructure featuring isolated closed pores. The rich morphologies generated upon sintering allows one to tune the thermal treatment to develop open or closed microporosity depending on the target application.


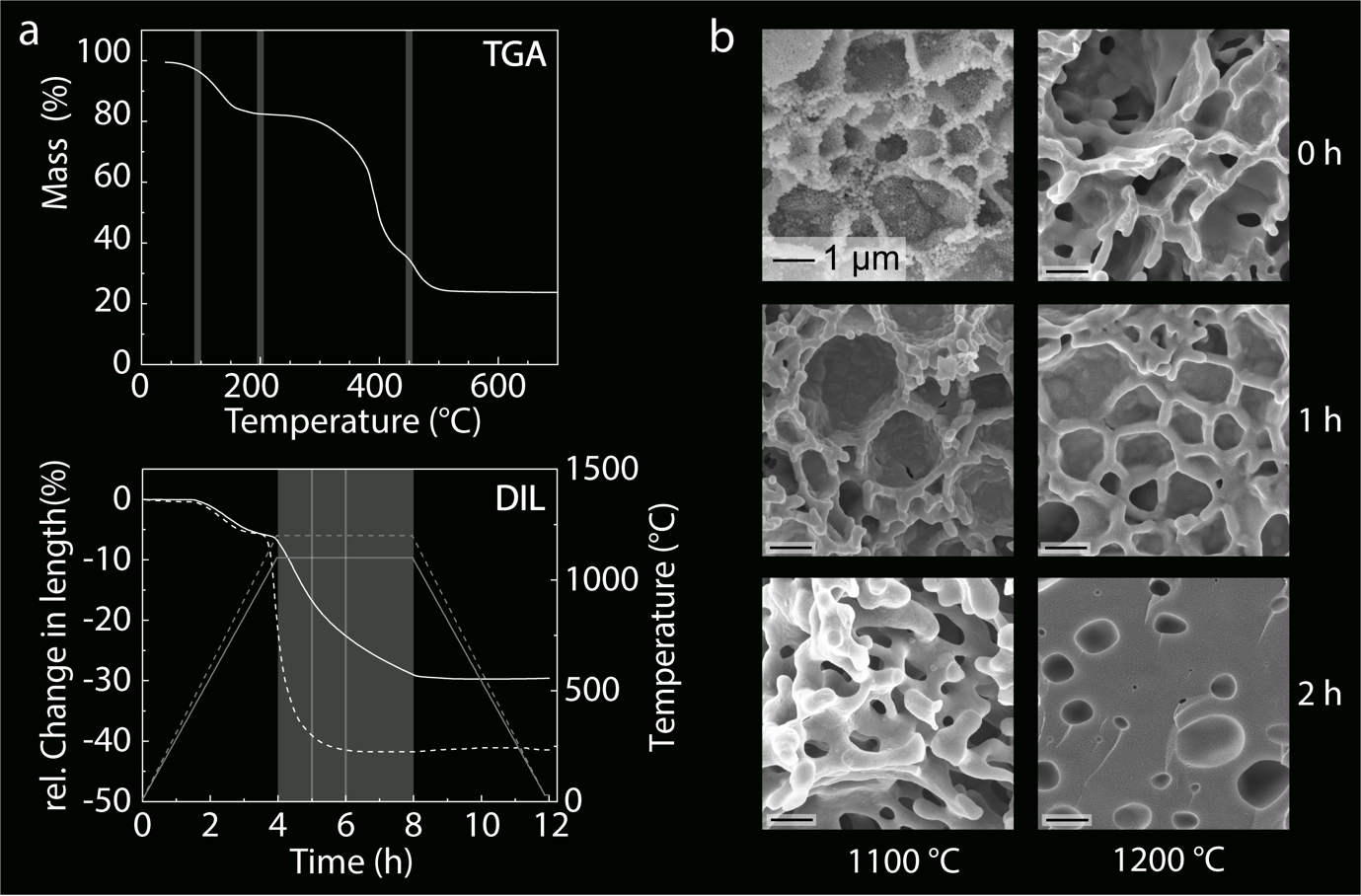


**Supplementary Figure 7.** Conversion of as-printed composites into inorganic hierarchical structures. (a) Thermogravimetric analysis (TGA) and dilatometry (DIL) of the as-printed composite, indicating the temperatures at which the organic phase is removed from the sample during calcination and the shrinkage of the material during sintering at high temperatures, respectively. The grey vertical lines in the TGA graph indicate the hold temperatures used for the calcination process. The shrinkage of calcined samples during sintering in shown in the DIL plot for heat treatments at 1100°C (full line) and 1200°C (dashed line). (b) SEM images depicting the microstructure of the struts after sintering at 1100 or 1200 °C for different elapsed times.

In addition to temperature and time, we also evaluated the effect of the water content of the initial emulsions on the sintering shrinkage of the structure. Measurements of the dimensions of printed Kelvin lattices before and after sintering reveal that the sintering shrinkage of the printed structures is relatively constant at 35-40% and is not dependent on the water content in the initial emulsion (Supplementary Figure 8). These results indicate that the sintering shrinkage is dominated by the continuous phase formed by particles and polymerized resin. Since the concentration of particles in the resin is constant, the shrinkage after sintering also remains unaltered regardless of the amount of dispersed water phase. This independence prevents cracking of graded structures obtained by stacking and sintering layers of polymerized emulsions with varying water contents (Supplementary Figure 9).


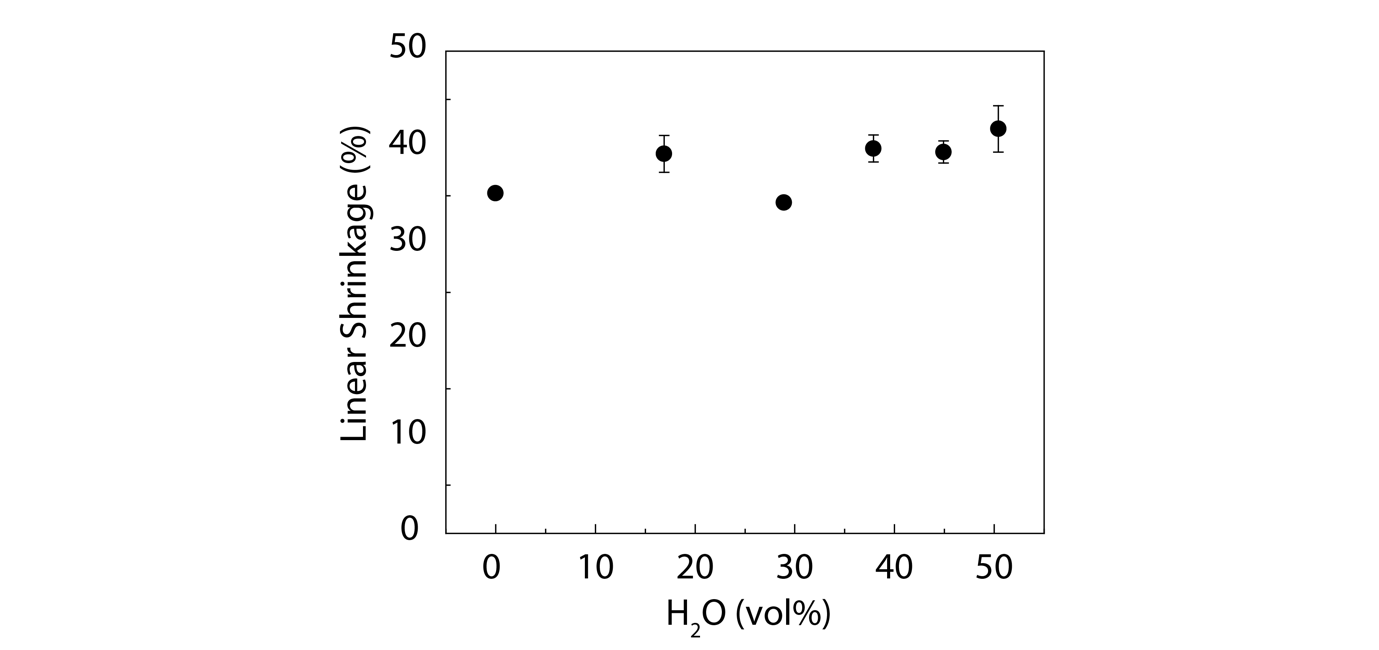


**Supplementary Figure 8.** Linear shrinkage of printed Kelvin lattices made from emulsions with water contents in the range 0 – 50 vol% after sintering at 1100 °C for 2h. Data refer to the average values from 1-3 samples measured along all three directions.

**SLA printed object with graded porosity**


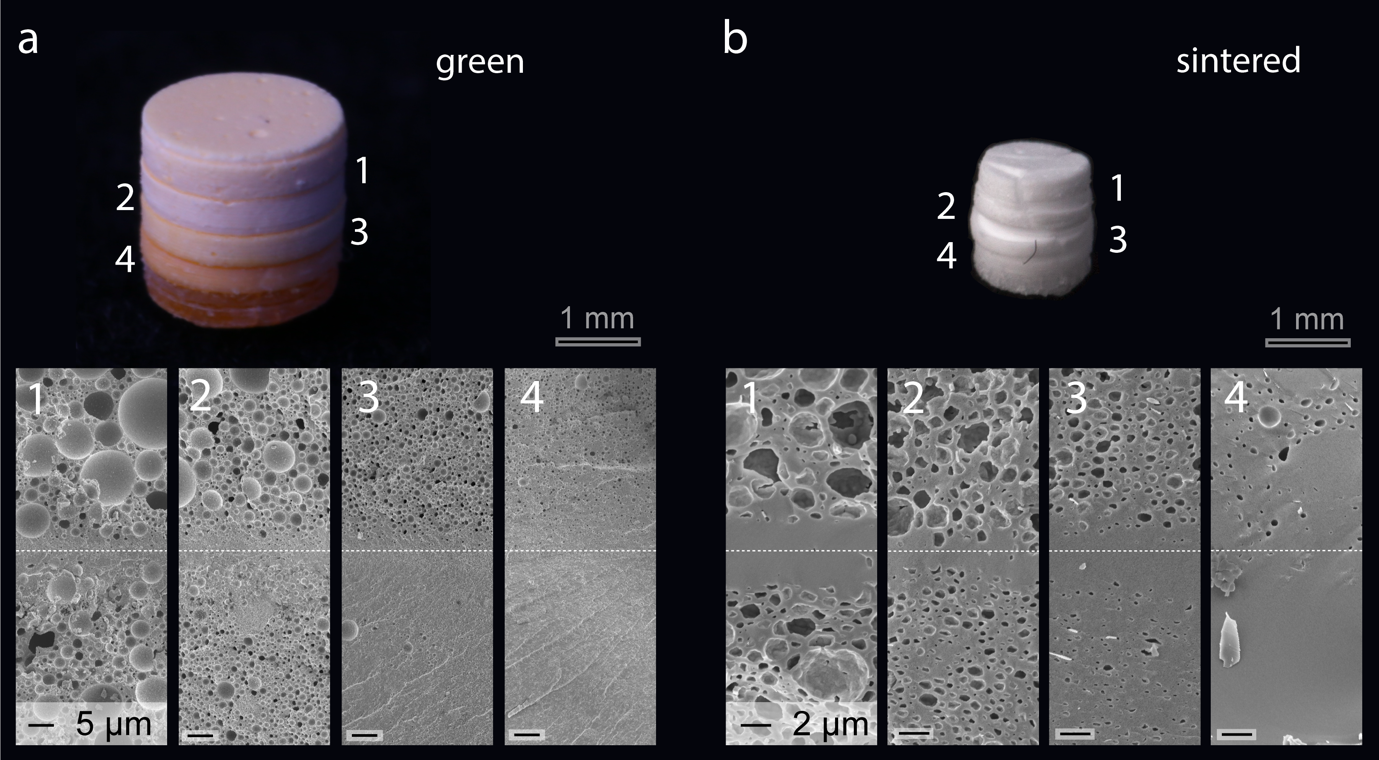


**Supplementary Figure 9.** Cylindrical-shaped objects with graded porosity before and after sintering. (a) As-printed (green) parts with graded porosity along the height prepared by varying the emulsion water content in individual polymerized layers. The numbers 1, 2, 3 and 4 indicate boundaries of the layers obtained using water contents in the ranges of 50-45 vol%, 29-45 vol%, 17-29 vol%, and 0-17 vol%, respectively. SEM close-ups of the interfaces between layers are also shown. (b) Sintered silica part with graded porosity along the height. SEM images depicting the microstructures of the graded cylinder-shaped object after sintering at 1200°C for 1 h. The graded porosity resulting from the increasing H_2_O content of the emulsion was preserved during the sintering process. Scale bar in (b): 2 µm**.**

**Adsorption properties of hierarchical porous structures**


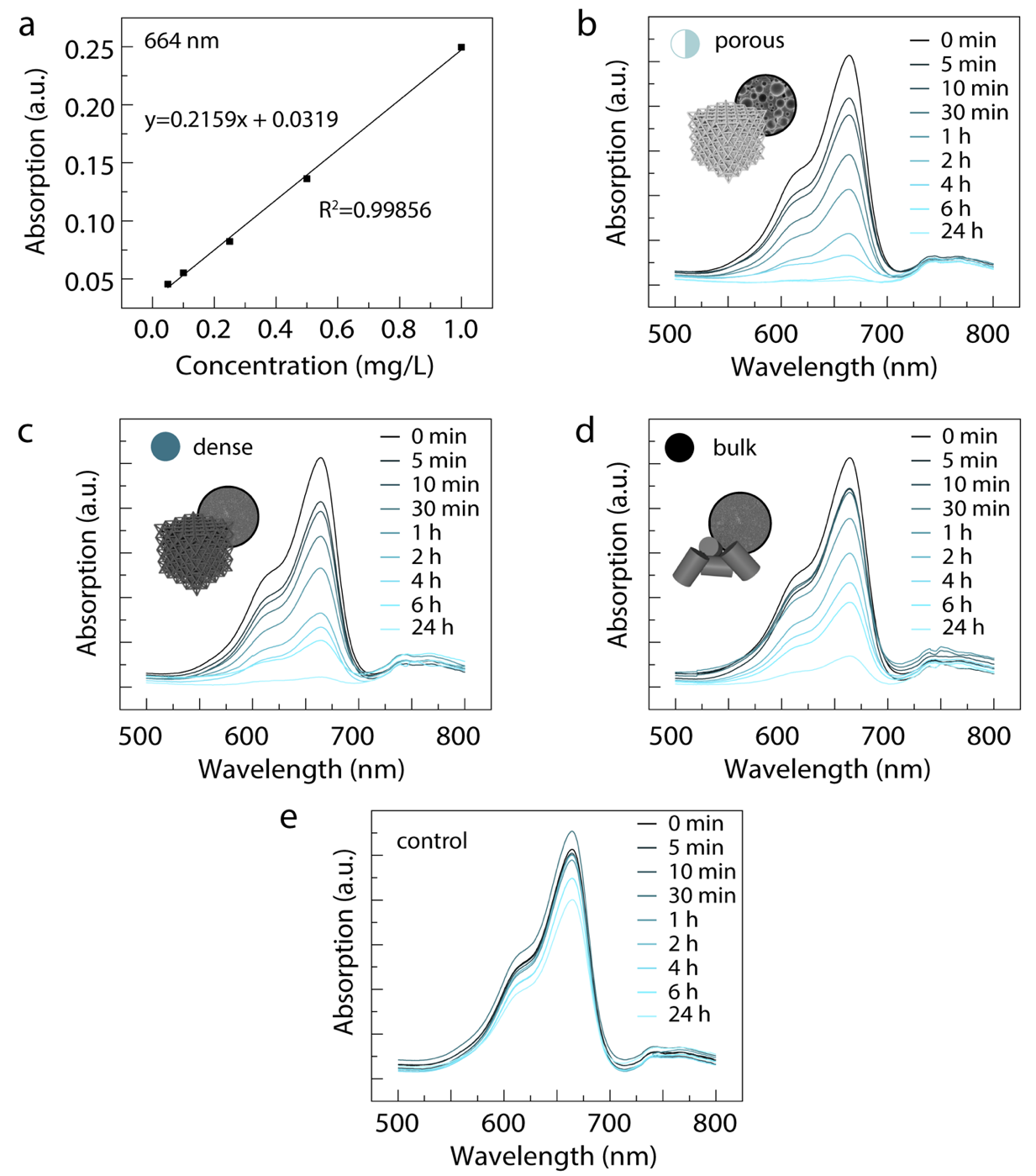


**Supplementary Figure 10.** Light absorption experiments performed to assess the adsorption performance of hierarchical porous structures (Figure 4c,d, main text). (a) Calibration curve correlating the light absorption at 664nm and the concentration of methylene blue in aqueous solution. (b-d) UV-Vis analysis of the MB aqueous solutions into which octet lattices with microporous (b) and dense struts (c) were immersed for distinct time periods. Plot (d) shows the data obtained for the reference bulk ceramic piece. 5mL of an aqueous solution with initial concentration of 10 mg/L methylene blue was used in all the experiments. The UV-Vis measurements show that the octet lattice with microporous struts has higher adsorption and is a factor eight faster than the bulk ceramic and twice as fast as the octet structure with dense struts over 24 hours of time. Control measurements without adsorbent are shown in (e).

**
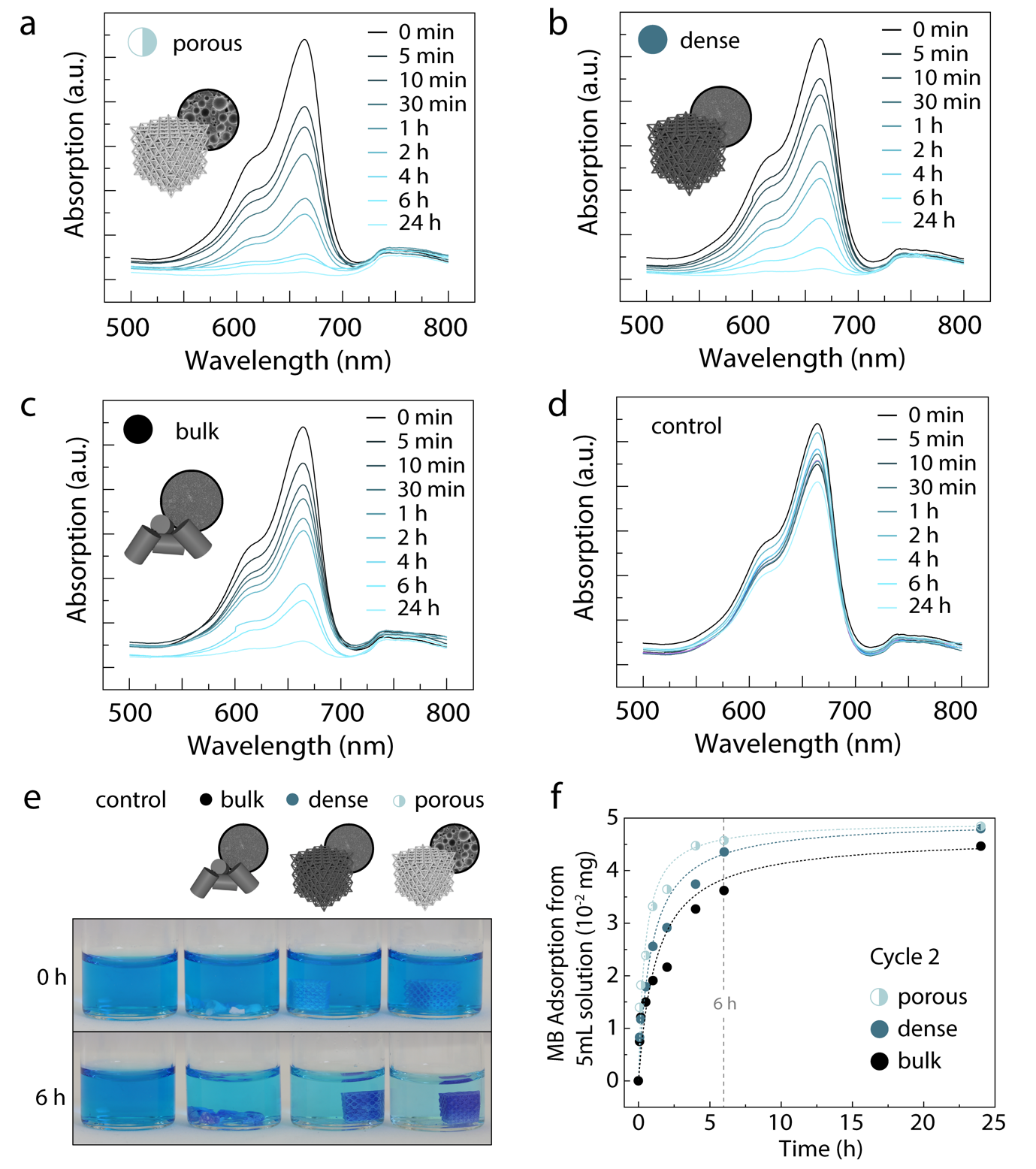
**

**Supplementary Figure 11.** Adsorption capacity of hierarchical porous structures that were recycled from the first experimental series (Figure 4c,d and Supplementary Figure 10). (a-c) UV-Vis analysis of the MB aqueous solutions into which octet lattices with (a) microporous and (b) dense struts were immersed for distinct time periods. A bulk ceramic piece was also tested for comparison (c). The second cycle of adsorption tests demonstrates that the structures can be recovered and reused at equal performance. Control measurements without adsorbent are shown in (e). 5 mL of an aqueous solution with initial concentration of 10 mg/L methylene blue was used in all the experiments.

**Pseudo-second order kinetic adsorption fitting of methylene blue adsorption**

The adsorption kinetics of the ceramic structures was quantified by fitting the experimental adsorption ($Q$ in mg) data with the following pseudo-second-order kinetics equation:

$$Q=\frac{Q_{e}k_{2}t}{Q_{e}k_{2}t+1}$$

where *k_2_* is a fitting parameter, *Q_e_* is the equilibrium adsorption in mg and *t* is the time of adsorption in hours.

The obtained fittings are shown as dashed lines in Figure 4d (adsorption cycle 1) and in the Supplementary Figure 11f (adsorption cycle 2).

**Surface area of as-printed and ceramic structures**

BET analysis was performed to quantify the surface area of octet lattices before (as-printed; green) and after sintering (Supplementary Figure 12). The lattices had a strut thickness of 0.5 mm and were sintered at 1100 °C for 2 h. The overall surface area clearly increases from 3.213 m^2^/g for dense, green samples, up to 18.687 m^2^/g for microporous, sintered samples. For green samples, the presence of water droplets was found to increase the nanoporosity by a factor of 2 compared to the droplet-free dense counterparts. These nanopores are probably formed during the transport of water through the continuous phase during the drying process. Furthermore, the surface area of microporous, sintered lattices is 50% higher than that of sintered lattices without microporosity. This increase in surface area explains the enhanced adsorption of molecules by the microporous sintered structure (Figure 4 and Supplementary Figures 10 and 11)


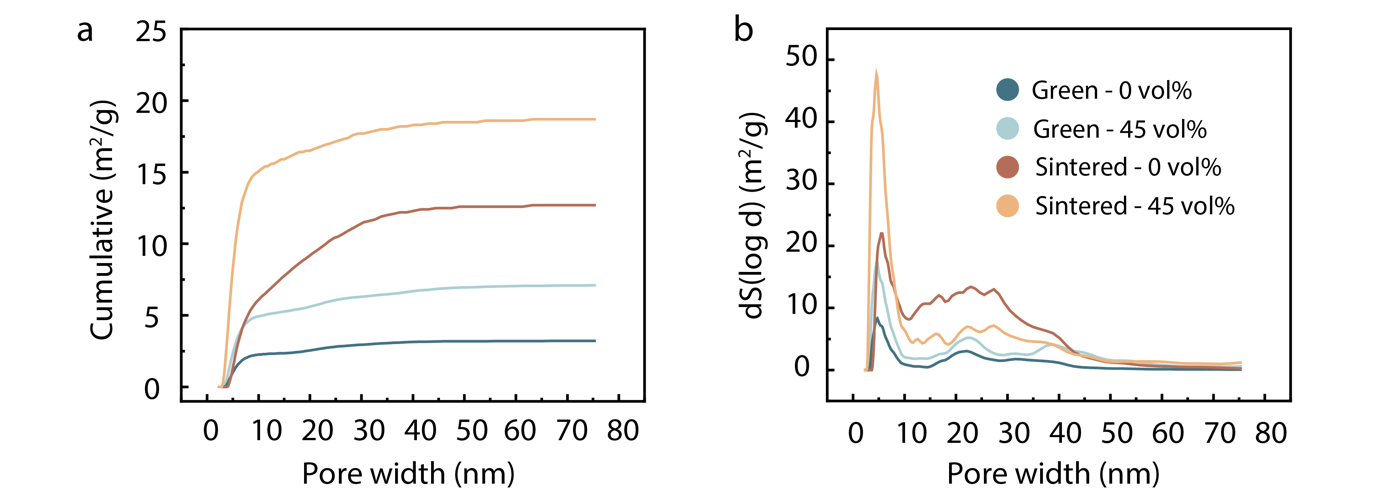


**Supplementary Figure 12.** Surface area of as-printed (green) and sintered octet lattices obtained from emulsions containing 0 or 45 vol% internal porosity. (a) Cumulative and (b) discrete values as a function of the pore size. Legend shown is identical for both graphs.
